# Anti-EGFR plus anti-PD1 in advanced and refractory cutaneous squamous cell carcinoma: a cohort study

**DOI:** 10.1093/oncolo/oyag080

**Published:** 2026-03-14

**Authors:** Camille Guy, Candice Hober, Sophie Maiezza, Philipe Jamme, Eve Desmedt, Laurent Mortier, Marie Boileau

**Affiliations:** Univ. Lille, CHU Lille, Service de Dermatologie, Lille, F-59000, France; Univ. Lille, CHU Lille, Service de Dermatologie, Lille, F-59000, France; Univ. Lille, CHU Lille, Service de Dermatologie, Lille, F-59000, France; Univ. Lille, CHU Lille, Service de Dermatologie, Lille, F-59000, France; Univ. Lille, CHU Lille, Service de Dermatologie, Lille, F-59000, France; Univ. Lille, CHU Lille, Service de Dermatologie, Lille, F-59000, France; Univ. Lille, Inserm, CHU Lille, U1189 - ONCO-THAI - Assisted Laser Therapy and Immunotherapy for Oncology, F-59000 Lille, France; Univ. Lille, CHU Lille, Service de Dermatologie, Lille, F-59000, France; Univ. Lille, Inserm, CHU Lille, U1189 - ONCO-THAI - Assisted Laser Therapy and Immunotherapy for Oncology, F-59000 Lille, France

**Keywords:** anti-EGFR, anti-PD1, advanced cutaneous squamous cell carcinoma

## Abstract

**Introduction:**

Advanced cutaneous squamous cell carcinoma (cSCC) is associated with a poor prognosis. Although anti-Programmed cell Death protein 1 (anti-PD1) immunotherapy has improved cSCC management, its efficacy remains limited in some patients. Second-line options, including anti–epidermal growth factor receptor (anti-EGFR) antibodies and chemotherapies, exhibit transient efficacy and often good tolerability. Given the lack of successive treatment lines, new strategies are emerging, such as the combination of anti-PD1 and anti-EGFR agents.

**Materials and Methods:**

We conducted a retrospective, monocentric cohort study including patients treated in our oncodermatology department between 2013 and 2024 for advanced cSCC refractory to anti-PD1 monotherapy and who received a combination of anti-PD1 and anti-EGFR as second- or third-line therapy. The aim was to assess the efficacy and safety of this combination.

**Results:**

Fourteen patients were included, predominantly male (12/14), with a median age of 63 years. Most tumors originated from head and neck primary sites (*n* = 8). Eleven patients had progressed after prior exposure to both anti-PD1 therapy and cetuximab combined with chemotherapy. Thirteen patients received cetuximab (500 mg/m^2^) combined with pembrolizumab every 3 weeks, and 1 received cetuximab with nivolumab. The overall response rate was 38.5%, including 15.5% complete and durable response after treatment discontinuation, observed at 9 and 16 months, and 23% partial responses. Responses occurred early, with subsequent deepening observed in 2 patients. Adverse events were mainly grade 1, and only 2 cases experienced grade 3 toxicity (acneiform rash).

**Conclusion:**

In a heavily pretreated real-life population with advanced cSCC, the anti-PD1/anti-EGFR combination showed clinical activity with acceptable safety, supporting its role as later-line strategy.

## Graphical abstract

**Graphical Abstract:**
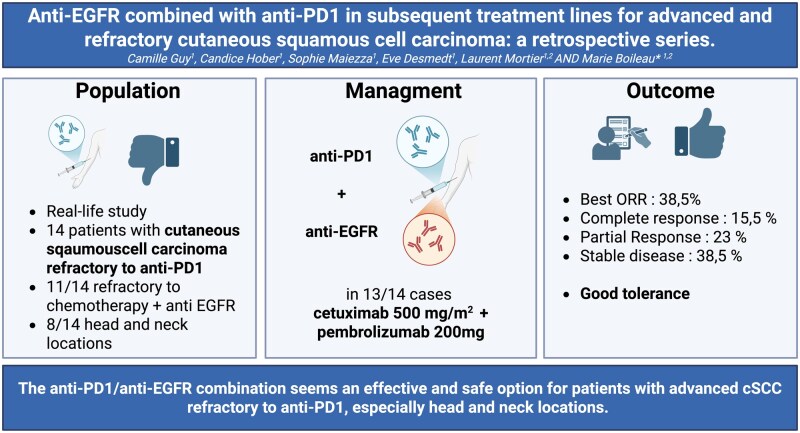

Implications for Practice This real-life study provides additional insight into the use of anti-EGFR and anti-PD1 combination therapy in a later-line setting, as most patients had already progressed after both anti-PD1 therapy and anti-EGFR combined with chemotherapy. In this heavily pretreated population, the combination demonstrated maintained clinical activity with an overall acceptable tolerability profile, including when administered using a reduced-intensity cetuximab dosing schedule adapted to elderly and frail patients. Responses were predominantly observed in tumors arising from head and neck primary sites, suggesting that tumor location may help inform clinical decision-making in this context. These findings complement existing prospective data largely focused on earlier lines of therapy and support consideration of this approach as a third-line option in selected patients when therapeutic options are limited.

## Introduction

Cutaneous squamous cell carcinoma (cSCC) is the second most common skin cancer, and it accounts for approximately 20% of all skin cancer cases.^1^^,^^2^ In France, the annual incidence of cSCC is estimated to be 30/100 000 people.^3^

In most cases, cSCC is diagnosed at an early stage; early-stage cSCC has a very favorable prognosis and can be managed by surgery alone. Overall survival at 10 years exceeds 90% after appropriate surgical management.^4^ However, approximately 5% of cases progress to advanced forms, which are no longer amenable to surgery or radiotherapy and require systemic treatment.^4^ These advanced forms include locally advanced cSCC (with deep invasion of muscle, nerve, or bone structures), cSCC with regional lymph node involvement affecting several drainage areas or localized near structures of functional or vital importance, and cSCC with distant metastases. In these situations, the prognosis is markedly poorer, making cSCC the second most common cause of cutaneous cancer–related death, with a median survival limited to 15.3 months after the start of first-line therapy.^5^

Today, immune checkpoint inhibitors targeting PD1 are considered the standard first-line systemic treatment for unresectable locally advanced or metastatic cSCC in most countries, based on several pivotal clinical trials demonstrating their efficacy.^6–10^ This position is further supported by the TOSCA study,^11^ a retrospective analysis showing a 43% reduction in the risk of death with cemiplimab compared with historical systemic treatments.

But in some patients, the efficacy of anti-PD1 agents remains inadequate, notably due to primary resistance (lack of initial response) or secondary resistance (therapeutic escape). Both are the result of mechanisms involving complex and constant interactions between tumor cells, the tumor microenvironment, and the immune system.

Currently, in the event of nonresponse to anti-PD1 therapy or poor tolerance, 2 second-line systemic therapeutic options are available: (1) anti–epidermal growth factor receptor (anti-EGFR)–targeted therapies, such as cetuximab, and (2) platinum-based chemotherapies. Radiotherapy is also effective for treating locally advanced cSCC. While these treatments have demonstrated some efficacy, their benefits are often limited owing to transient responses and modest tolerability, particularly in patients who are often frail due to their advanced age and multiple comorbidities.^12^

**Figure 1.**
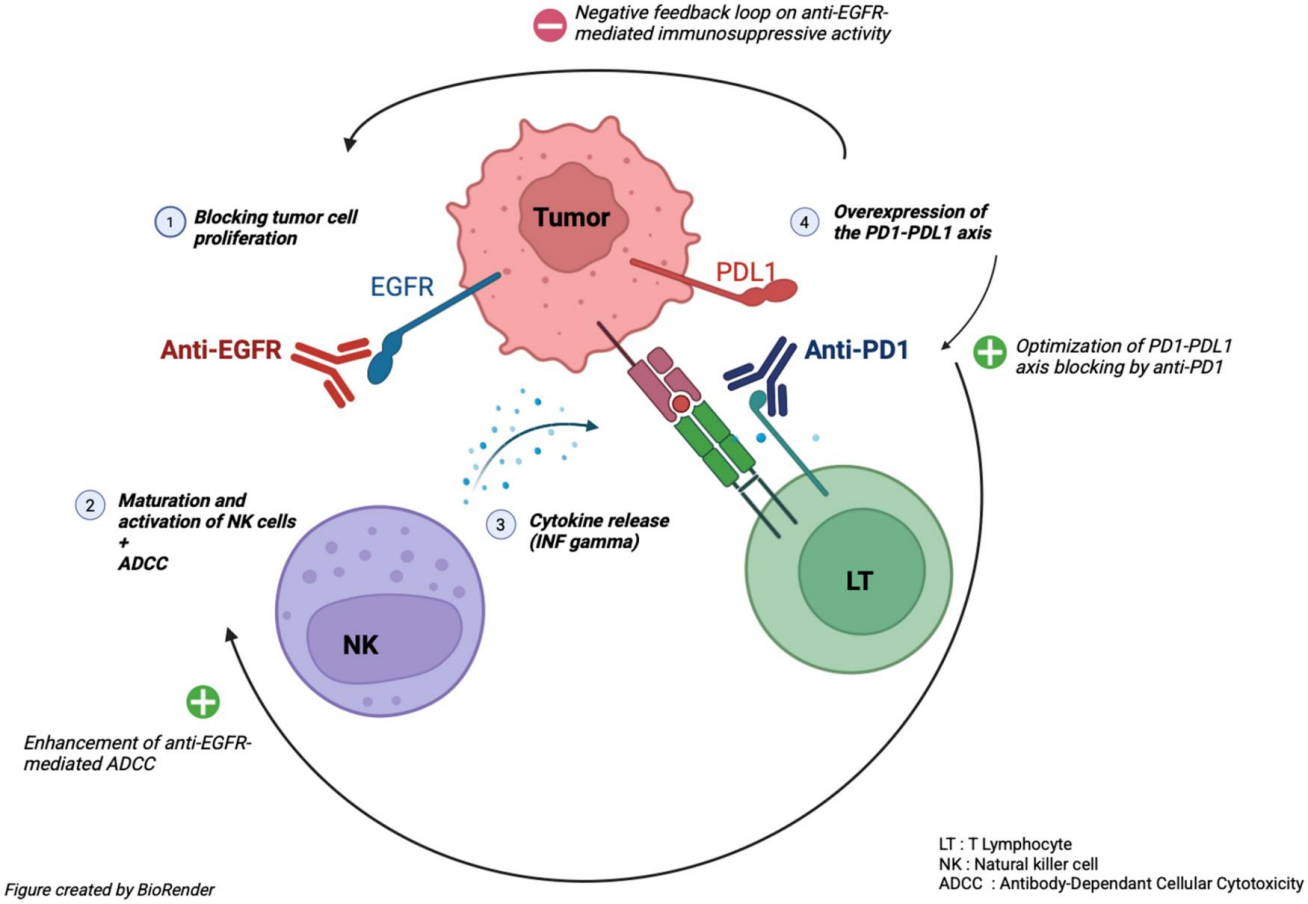
Synergistic effect of anti-EGFR agent and anti-PD1 agent. EGFR: Epithelial Growth Factor Receptor; anti-PD1: anti-Programmed cell Death protein 1; anti-PDL1: anti-Programmed cell Death protein Ligand 1; INF Gamma: interferon gamma.

In this context, new therapeutic strategies are emerging with the aim of increasing the efficacy of anti-PD1 treatments and overcoming resistance mechanisms. Among these promising approaches, the combination of anti-PD1 therapy and anti-EGFR therapy appears to be a particularly interesting option.^13^^,^^14^ The EGFR, which is located on cell surfaces, is overexpressed in many cancers, leading to uncontrolled cell proliferation. Anti-EGFR agents, such as cetuximab, are monoclonal antibodies that specifically bind to the extracellular domain of EGFR, thereby limiting this abnormal cancer cell proliferation. In addition, anti-EGFR agents are capable of stimulating an antitumor immune response by activating Natural Killer (NK) cells and then promoting the release of cytokines, notably interferon gamma, which induces the overexpression of PD1 and / or it ligand. Paradoxically, this cytotoxic cell activation results in a negative feedback loop of immunosuppressive activity, limiting the efficacy of anti-EGFR agents. However, blockade of the PD1–PDL1 axis by immune checkpoint inhibitors is thus optimized by this overexpression, which also increases anti-EGFR–mediated antibody-dependent cytotoxic activity and thus overcomes this dysfunction (Figure 1).

Furthermore, the favorable tolerability of anti-PD1 and anti-EGFR compared with conventional chemotherapies is a significant advantage, particularly in this patient population that is often vulnerable.

Although many studies on first-line treatments for advanced cSCC are available, data and therapeutic options for subsequent treatment lines are lacking.

We conducted a retrospective cohort study under real-life conditions to evaluate the efficacy and tolerability of the combination of anti-PD1 therapy and anti-EGFR therapy in subsequent treatment lines for the management of advanced cSCC refractory to anti-PD1 therapy.

## Methods

We conducted a retrospective, monocentric cohort study. We included all patients who were managed in our oncodermatology department between 2013 and 2024, who presented with advanced cSCC, and who received a combination of anti-EGFR and anti-PD1 treatments that were administered as second- or third-line therapy after the failure of anti-PD1 monotherapy. No exclusion criteria were applied.

The primary objective was to descriptively evaluate the efficacy and safety of the combination of anti-EGFR and anti-PD1 agents in the treatment of advanced cSCC refractory to anti-PD1 therapy. Patients were identified using the pharmacy prescription register. All cases were reviewed and validated during a multidisciplinary tumor board meeting. We reviewed all the medical reports available in our hospital management software. Adverse events were classified according to the applicable version of the Common Terminology Criteria for Adverse Events (CTCAE v5.0). All the patients underwent extensive imaging assessments at regular intervals of approximately 3 months via computed tomography, magnetic resonance imaging, or positron emission tomography scan, depending on the initial location of the tumor and its extension in the standard of care. Progression was assessed according to the RECIST 1.1 criteria.

The collected data included age, sex, tumor location, previous systemic treatments for cSCC, treatment with a regimen combining anti-EGFR and anti-PD1, and follow-up data on its efficacy and tolerance. Approval was obtained from the Data Protection Office in accordance with current regulations and local ethical guidelines (N/Ref: DEC25-061).

## Results

We included 14 patients whose characteristics are described in Table 1. The population included 12 men and 2 women, with a median age of 63 years (min-max: 45-85). Eight patients had lesions in the head and neck region, 3 patients had lesions in the limbs, 2 patients had lesions in the trunk, and 1 patient had lesions in the penis. Among these patients, 2 had locally advanced cSCC, 9 had lymph node involvement, and 3 had visceral metastases. One patient was not evaluable because he received only a single course of treatment due to deterioration in his general condition that was unrelated to the treatment.

**Table 1.** Population characteristics

Population characteristics	Number
**Number of patients, *n***	14
**Male, *n***	12
**Median age, years (min-max)**	63 (45-85)
**Location**	
**Head and neck**	8
**Other location**	6
**Limb(s)**	3
**Trunk**	2
**Penis**	1
**Stage**	
**Locally advanced**	2
**Lymph node involvement**	9
**Visceral metastases**	3
**Previous systemic treatments received**	
**Anti-PD1 (cemiplimab, pembrolizumab, or nivolumab)**	14
**Anti-EGFR (cetuximab) + chemotherapy (carboplatin)**	11
**Anti-PD1 + anti-EGFR combination**	
**Cetuximab + pembrolizumab/3 weeks (C1 = D21)**	13
**Cetuximab + nivolumab/4 weeks (C1 = D28)**	1

Anti-PD1, Anti-Programmed cell Death protein 1; Anti-EGFR, Anti-Epithelial Growth Factor Receptor.

All the patients had previously received systemic anti-PD1 monotherapy (cemiplimab, pembrolizumab, or nivolumab), and 11 of 14 had been treated with a combination of cetuximab and carboplatin, in most cases as first-line therapy. Thirteen patients received a regimen combining cetuximab (500 mg/m^2^) and pembrolizumab (200 mg), administered every 21 days. Only 1 patient was treated with cetuximab (500 mg/m^2^) and nivolumab (480 mg), administered every 28 days due to a specific request from the pharmacy (genital location).

The median follow-up was 12 months (IQR: 8-28). The median duration of anti-PD1 and anti-EGFR treatment was 7 months (IQR: 4-14).

The overall response rate at 3 months was 38.5%. The best overall response rate was 38.5%, which included 15.5% of patients with a complete response and 23% of patients with a partial response (Table 2). The median time to complete response was 12.5 months, whereas the median time to progression was 5.5 months (IQR: 3-9). Three patients died due to cSCC progression.

**Table 2.** Response and disease control rates.

Variable	Response at 3 months		Best overall response	
	*N* = 13	%	*N* = 13	%
**Complete response (CR)**	0	0	2	15.5
**Partial response (PR)**	5	38.5	3	23
**Stable disease (SD)**	5	38.5	5	38.5
**Progression**	3	23	3	23
**Overall response rate—ORR (CR + PR)**	–	38.5	–	38.5
**Control rate (CR + PR + SD)**	–	77	–	77

**Figure 2.**
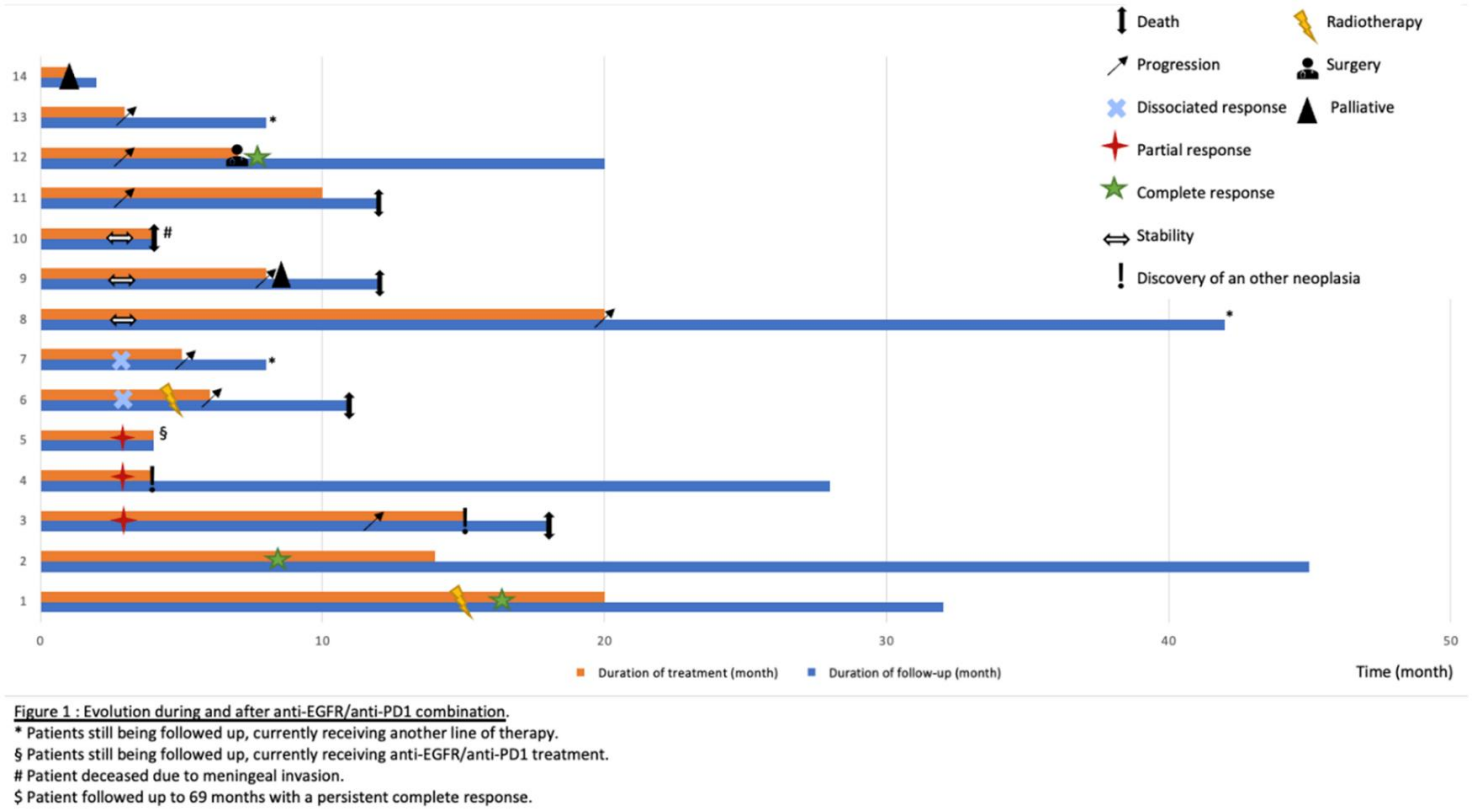
Progression during and after anti-EGFR/anti-PD1 combination therapy.

**Figure 3.**
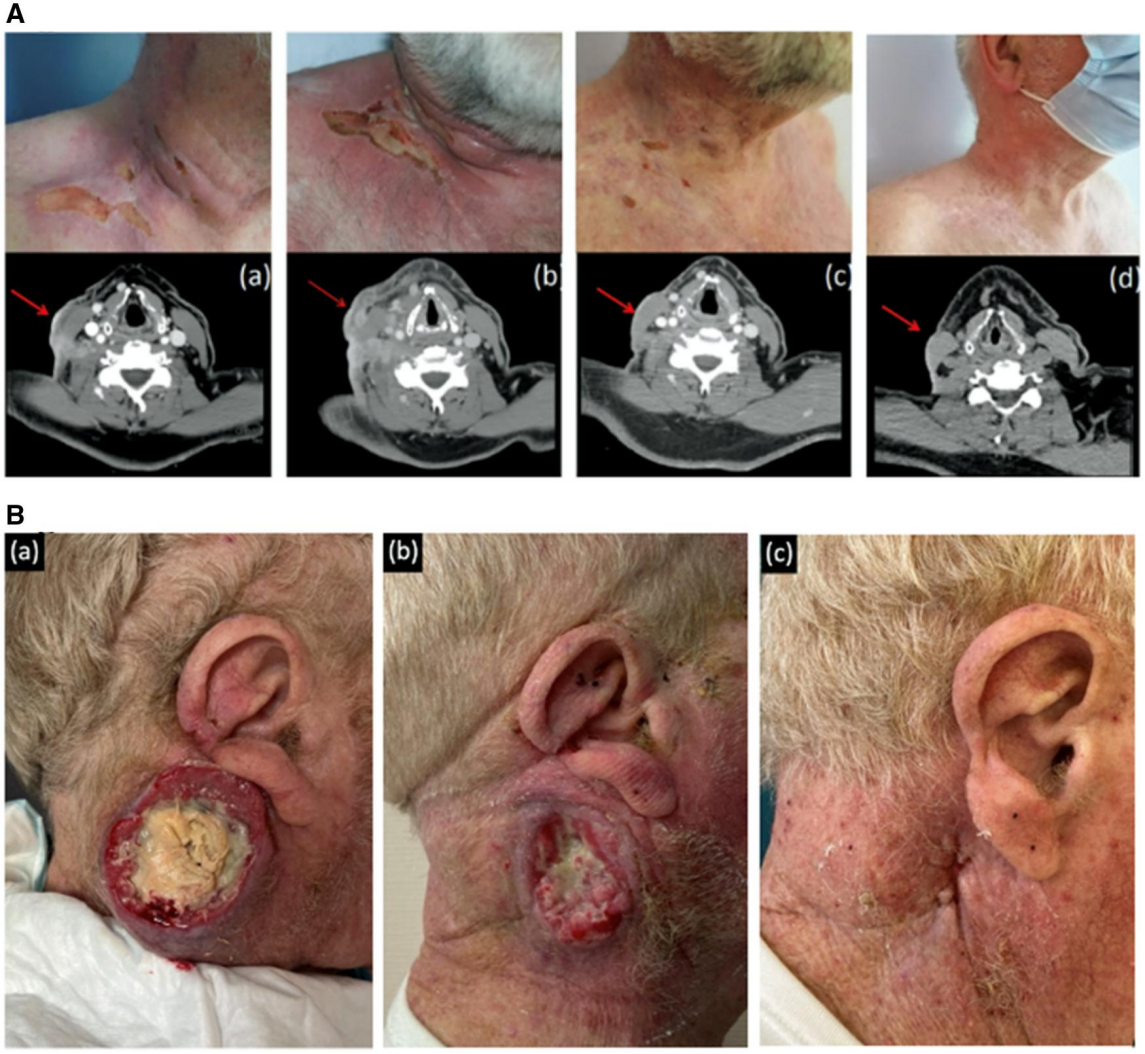
(A) Sequential clinical photographs of a patient with cutaneous squamous cell carcinoma showing a complete response achieved at 9 months after initiation of anti-PD1 and anti-EGFR combination therapy, with persistent response at 69 months of follow-up. (a) Before the start of pembrolizumab, (b) after 4 cycles of pembrolizumab, (c) after 3 months, and (d) 2 years of treatment with pembrolizumab/cetuximab. (B) Sequential clinical photographs of a patient with cutaneous squamous cell carcinoma demonstrating a partial response to anti-PD1 and anti-EGFR combination therapy, with ongoing treatment at the time of data cutoff. (a) Before treatment initiation, (b) after 1 cycle, and (c) after 3 cycles of pembrolizumab/cetuximab.

Treatment outcomes are summarized in Figure 2. Two patients (15%) experienced a durable complete response at 16 and 9 months, with a persistent response duration of 12 and 69 months at last follow-up (Figure 3A). Partial responses were observed in 3 patients (23%), including 1 patient who was still receiving treatments at the time of data cutoff (Figure 3B). In the other 2 patients who achieved a partial response, other cancers with a worse prognosis (colon cancer and esophageal and laryngeal squamous cell carcinoma) were discovered, leading to a change in management. In 1 patient, the staging workup revealed a persistent lymph node response at the initial cSCC drainage site after treatment had stopped. Five patients (36%) had stable disease at 3 months, followed by delayed progression for 2 of these patients after 9 and 21 cycles, respectively. Two patients showed progression after 6 months. The last patient died quickly following meningeal invasion of his cSCC. Finally, disease progression was observed from treatment initiation in 3 patients (23%). Among the 11 patients previously exposed to both anti-PD1 therapy and cetuximab, the objective response rate was 36.4%, including 2 complete and 2 partial responses; 2 dissociated responses, 2 stable diseases, and 3 progressions were also observed.

We observed well-known immune-related adverse events and anti-EGFR–related toxicities, such as acneiform rashes (9 patients, 64%), plantar fissures (3 patients, 21%), hypomagnesemia (3 patients, 21%), lymphopenia (3 patients, 21%), and immune-induced corticotropic insufficiency (3 patients, 21%). Most of these toxicities were grade 1 toxicities (85%), with only 2 cases of grade 3 acneiform rash (15%). However, no adverse events required treatment interruption or dose reduction.

## Discussion

This retrospective real-life cohort study provides descriptive data on the use of anti-PD1 and anti-EGFR combination therapy in a later-line setting for patients with advanced and refractory cSCC. In a heavily pretreated population characterized by advanced age, clinical frailty, and multiple prior treatment failures, an objective response rate of 38.5% was observed, with an overall acceptable tolerability profile.

Tumors predominantly arose from head and neck primary site, and notably, all complete responses were observed in this subgroup. This observation suggests that tumor location may influence sensitivity to combined anti-PD1 and anti-EGFR therapy. However, this finding remains hypothesis-generating and should be interpreted cautiously given the limited sample size.

Interestingly, the overall response rate observed at 3 months was identical to the best overall response rate during follow-up, indicating that objective responses to the anti-EGFR/anti-PD1 combination occurred early after treatment initiation. Nonetheless, response depth evolved over time, with 2 patients experiencing a conversion from partial to complete response. This pattern suggests response maturation rather than delayed recruitment of new responders and is biologically plausible with the proposed synergistic interactions between EGFR inhibition and immune checkpoint blockade.

These findings are consistent with the limited data available in the literature. In a case series reported by Hsu et al.,^15^ 3 complete responses were observed in patients who had progressed on anti-PD1 monotherapy after the addition of panitumumab, another anti-EGFR agent. Prior to combination therapy, patients had received 2, 5, and 7 cycles of anti-PD1 monotherapy, respectively. The combination was then continued for 27, 7, and 5 cycles, respectively, leading to complete and durable responses in all cases. Similarly, Chen et al.^16^ reported a complete response after 6 months of first-line cetuximab plus nivolumab in a patient with locally advanced cSCC of the ear who was ineligible for surgical management.

Within this therapeutic continuum, our study builds upon prior sequencing data by addressing a subsequent, later-line clinical scenario. In a retrospective single-institution study, Marin-Avecedo et al.^17^ reported high response rates with cetuximab monotherapy when administered immediately after anti-PD1 failure (64%), whereas no responses were observed when cetuximab was introduced after an intervening line of therapy. Although this study did not evaluate combination therapy, it highlighted the importance of treatment sequencing in an advanced cSCC.

More recently, prospective studies have further clarified the role of anti-EGFR and anti-PD1 strategies across different disease settings. The I-TACKLE trial^18^ demonstrated that adding cetuximab to pembrolizumab at the time of progression could restore antitumor activity in patients with primary or secondary resistance to anti-PD1 therapy, achieving an objective response rate of 44% (10/23). This supports the biological relevance of EGFR inhibition as a salvage strategy following immunotherapy failure.

In contrast, the randomized phase II Alliance A091802 trial^19^ addressed an earlier disease setting, evaluating the upfront combination of anti-PD1 and anti-EGFR therapy in immunotherapy-naïve patients. Although the combination significantly improved progression-free survival (PFS) compared with anti-PD1 alone (median PFS 11.1 vs 3.0 months; Hazard Ratio 0.48), the increase in objective response rate was modest (27.6% vs 21.4%) and accompanied by a substantially higher incidence of grade ≥3 adverse events (48.3% vs 21.5%), highlighting tolerability as a key limiting factor of first-line use.

Taken together, these prospective data confirm that anti-PD1/anti-EGFR combinations are biologically active across different lines of therapy. Our results are concordant with these observations, showing a comparable overall response rate in a real-life cohort.

Importantly, further analyses of our population revealed a particularly refractory clinical context. Although the study initially focused on patients previously exposed to anti-PD1 therapy, most evaluable patients (11/13) had in fact already progressed after both anti-PD1 therapy and anti-EGFR–based chemotherapy, corresponding to a later-line situation that is frequently encountered in routine practice but remains underrepresented in clinical trials. Despite this unfavorable context, an objective response rate of 36.4% (4/11) was observed in this subgroup, including 2 complete and 2 partial responses, suggesting that the combination may retain clinically meaningful activity even after sequential failure of both therapeutic classes.

An additional original aspect of our study is the use of an alternative cetuximab dosing schedule. To reduce treatment burden and hospital visits in an elderly and frail population, cetuximab was administered at 500 mg/m^2^ every 3 weeks, differing from standard regimens (250 mg/m^2^ weekly or 500 mg/m^2^ biweekly), and not previously evaluated in this setting. Despite this adapted schedule, clinical activity was maintained with acceptable tolerability, supporting its feasibility in routine practice for patients requiring long-term treatment.

Overall, our findings provide real-life evidence supporting the feasibility and clinical activity of the anti-PD1 and anti-EGFR combination therapy in advanced cSCC. Beyond confirming signals reported in prospective studies, our work adds original data in a routine practice setting, including patients treated in later lines after multiple therapeutic failures.

Several limitations must be acknowledged. The retrospective, single-center design exposes the analysis to selection and information biases and precludes causal inference regarding treatment efficacy. The small sample size limits statistical power and restricts subgroup analyses to an exploratory level. In addition, heterogeneity in disease extent, prior systemic treatments, and treatment sequencing reflect real-life practice but limit direct comparability between patients. The absence of a control group further prevents formal assessment of the relative contribution of the combination compared with alternative strategies. Finally, variable follow-up may have limited the detection of late toxicities or delayed responses. Taken together, these limitations warrant cautious interpretation of our results, which nonetheless provide meaningful real-life evidence on the feasibility, tolerability, and potential activity of anti-PD1 and anti-EGFR combination therapy in a heavily pretreated patient population. Future studies should focus on optimizing treatment sequencing, dosing schedules, and patient selection.

## Conclusion

Although limited by its retrospective, single-center design and small sample size, this real-life study provides additional insight into the use of anti-PD1 and anti-EGFR combination therapy in patients with advanced cSCC who have received multiple prior systemic treatments. In this heavily pretreated population, the combination showed meaningful clinical activity with an overall acceptable safety profile, including in elderly patients and in tumors arising from head and neck primary tumor sites. These findings are consistent with previously reported data and add original real-life evidence in later-line settings that remain underrepresented in clinical trials. Our results support the clinical relevance of this strategy when therapeutic options are limited. Further prospective studies are warranted to better define the optimal treatment line and clinical context in which this combination may be most appropriately integrated into the management of an advanced cSCC.

## Data Availability

The data supporting the findings of this study are available from the corresponding author upon reasonable request.
